# Supplementary material for: Lymph node metastasis prediction of papillary thyroid carcinoma based on transfer learning radiomics
Source: Nat Commun. 2020 Sep 23;11:4807. doi: 10.1038/s41467-020-18497-3 (PMC7511309; doi:10.1038/s41467-020-18497-3)
Supplement: Supplementary file 1 — Supplementary Information [file 41467_2020_18497_MOESM1_ESM.pdf]

Supplementary Information

# Lymph node metastasis prediction of papillary thyroid carcinoma based on transfer learning radiomics

Jinhua Yu et al.

Supplementary Table 1. Quantitative indexes of machine experimental results

| Machine | AUC | ACC | SENS | SPEC | PPV | NPV | MCC | F1score |
|---|---|---|---|---|---|---|---|---|
| GE | 0.91 | 0.83 | 0.86 | 0.81 | 0.77 | 0.89 | 0.66 | 0.81 |
| Kretztechnik | 0.87 | 0.79 | 0.89 | 0.73 | 0.70 | 0.90 | 0.61 | 0.78 |
| SuperSonic | 0.84 | 0.82 | 0.82 | 0.81 | 0.69 | 0.90 | 0.61 | 0.75 |

Supplementary Table 2. Quantitative indexes of doctor experimental results

| Doctor | AUC | ACC | SENS | SPEC | PPV | NPV | MCC | F1score |
|---|---|---|---|---|---|---|---|---|
| 1 | 0.92 | 0.84 | 0.84 | 0.84 | 0.70 | 0.92 | 0.65 | 0.76 |
| 2 | 0.87 | 0.84 | 0.74 | 0.92 | 0.90 | 0.80 | 0.68 | 0.81 |
| 3 | 0.91 | 0.90 | 0.83 | 0.94 | 0.86 | 0.92 | 0.77 | 0.84 |

Supplementary Table 3. Quantitative index comparisons of VGG, ResNet, Inception ResNet, and TLR on three cohorts

| Method | AUC | ACC | SENS | SPEC | PPV | NPV | MCC | F1score |
|---|---|---|---|---|---|---|---|---|
| ***Testing set of the main cohort*** | | | | | | | | |
| VGG | 0.77 | 0.74 | 0.65 | 0.80 | 0.69 | 0.78 | 0.46 | 0.67 |
| ResNet | 0.77 | 0.71 | 0.47 | 0.87 | 0.70 | 0.71 | 0.37 | 0.56 |
| InceptionResNet | 0.75 | 0.71 | 0.46 | **0.89** | 0.73 | 0.71 | 0.39 | 0.56 |
| Our model | **0.93** | **0.84** | **0.94** | 0.77 | **0.73** | **0.95** | **0.69** | **0.82** |
| ***Independent testing set 1*** | | | | | | | | |
| VGG | 0.58 | 0.55 | 0.33 | 0.86 | 0.77 | 0.47 | 0.22 | 0.46 |
| ResNet | 0.58 | 0.55 | 0.34 | 0.85 | 0.77 | 0.47 | 0.22 | 0.47 |
| InceptionResNet | 0.56 | 0.56 | 0.45 | 0.71 | 0.69 | 0.47 | 0.16 | 0.55 |
| Our model | **0.93** | **0.86** | **0.83** | **0.89** | **0.92** | **0.78** | **0.71** | **0.87** |
| ***Independent testing set 2*** | | | | | | | | |
| VGG | 0.66 | 0.65 | 0.59 | 0.70 | 0.59 | 0.70 | 0.29 | 0.59 |
| ResNet | 0.59 | 0.61 | 0.55 | 0.65 | 0.54 | 0.66 | 0.20 | 0.54 |
| InceptionResNet | 0.64 | 0.63 | 0.43 | **0.77** | 0.58 | 0.65 | 0.21 | 0.49 |
| Our model | **0.93** | **0.84** | **0.95** | 0.75 | **0.74** | **0.96** | **0.70** | **0.83** |

Supplementary Table 4. Results of the ablation experiments.

| **Transfer learning (T) and hyperparametric optimization (H)** | **AUC value for the comparison of results** | | |
|---|---|---|---|
| | ***Testing set of the main cohort*** | ***Independent testing set 1*** | ***Independent testing set 2*** |
| T -, H - | 0.718 | 0.604 | 0.614 |
| T +, H - | 0.791 | 0.650 | 0.594 |
| T -, H + | 0.817 | 0.808 | 0.792 |
| T +, H + | 0.927 | 0.928 | 0.932 |

Supplementary Table 5. Information of machine used to collect the main cohort

| No. | Machine | Total number | Proportion |
|---|---|---|---|
| **1** | **GE Healthcare** | 206 | 20.34% |
| **2** | **SuperSonic Imagine SA** | 130 | 12.83% |
| **3** | **Kretztechnik** | 107 | 10.56% |
| **4** | Philips Medical Systems | 93 | 9.18% |
| **5** | TOSHIBA_MEC | 83 | 8.19% |
| **6** | GE Healthcare Austria GmbH & Co OG | 78 | 7.70% |
| **7** | MEDISON | 78 | 7.70% |
| **8** | SIEMENS | 74 | 7.31% |
| **9** | ESAOTE | 63 | 6.22% |
| **10** | TOSHIBA_MEC_US | 55 | 5.43% |
| **11** | Philips Healthcare | 37 | 3.65% |
| **12** | SAMSUNG MEDISON CO.,LTD. | 5 | 0.49% |
| **13** | Ultrasonix Medical Corp. | 2 | 0.20% |
| **14** | GE Vingmed Ultrasound | 1 | 0.10% |
| **15** | MINDRAY | 1 | 0.10% |
| **Total** | | 1013 | 100.0% |

Supplementary Table 6. Information about sonographers involved in collecting the main cohort

| No. | Year of experience | Collected cases | Proportion |
|---|---|---|---|
| **1** | 10 | 98 | 9.67% |
| **2** | 5 | 73 | 7.21% |
| **3** | 16 | 72 | 7.11% |
| 4 | 30 | 66 | 6.52% |
| 5 | 10 | 66 | 6.52% |
| 6 | 35 | 59 | 5.82% |
| 7 | 18 | 57 | 5.63% |
| 8 | 8 | 54 | 5.33% |
| 9 | 40 | 53 | 5.23% |
| 10 | 13 | 48 | 4.74% |
| 11 | 10 | 47 | 4.64% |
| 12 | 10 | 47 | 4.64% |
| 13 | 40 | 46 | 4.54% |
| 14 | 30 | 43 | 4.24% |
| 15 | 8 | 42 | 4.15% |
| 16 | 15 | 38 | 3.75% |
| 17 | 10 | 36 | 3.55% |
| 18 | 13 | 20 | 1.97% |
| 19 | 45 | 19 | 1.88% |
| 20 | 5 | 13 | 1.28% |
| 21 | 35 | 12 | 1.18% |
| 22 | 5 | 4 | 0.39% |
| Total | | 1013 | 100.0% |

Supplementary Note 1. Clinical Statistical Model

The Lasso regression model for LNM prediction was established as following.

$$\hat{y} = 0.0951x_1 - 0.0097x_2 - 0.0017x_3 + 0.0002319x_4 - 0.0001548x_5 - 0.0000635x_6 + 0.02x_7 - 0.0594x_8 + 0.019x_9 + 0.5722$$

where $x_1$, $x_2$, $x_3$, $x_4$, $x_5$, $x_6$, $x_7$, $x_8$ and $x_9$ denote the quantitative values for gender, age, TSH, TG, TGAb, TPOAB, tumor size, Hashimoto's disease and micro-calcification, respectively.

Supplementary Note 2. Traditional Radiomics Model

The 614 high-throughput features used in our study can be summarized in Supplementary Table 7:

Supplementary Table 7. Summary of high-throughput features

| Feature Type | Number of features | Quantitative Description |
|---|---|---|
| Demographic information | 2 | Age, Gender |
| Size | 3 | Diameter of an equivalent circle, Area, Ratio of tumor area to its convex hull |
| Shape | 3 | Radio of convexity to tumor perimeter, compactness, Rectangle-fitting factor |
| Margin & Boundary | 13 | Speculation, Extreme points of radial distance, Lobule number, Moment difference, Edge roughness, Acutance1, Acutance 2, Local window mean, Ratio of deviation inside and outside of tumor (In vs. Out), Mean contrast correlation coefficient of In vs. Out, Standard deviation of In vs. Out, Standard deviation (SD) of the annular region, Signal-to-noise ratio of the annular region |
| Orientation | 3 | Elliptical-normalized eccentricity, Elliptical-normalized angle, Length-to-width ratio |
| Position | 7 | 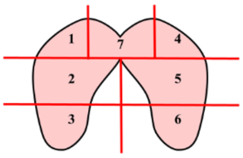 |
| | 3 (Relative position) | Overlap length with thyroid gland, Overlap area to tissue outside of thyroid gland, Distance to thyroid capsule |
| Echo pattern | 11 (Echo intensity) | Mean tumor contrast, Mean tumor covariance, Mean tumor non-similarity, Mean contrast correlation coefficient, Deviation ratio of the tumor tissue and normal thyroid gland, Relative brightness of the tumor and normal tissue, Relative brightness of tumor and normal muscle, SD of tumor contrast, SD of tumor covariance, SD of tumor non-similarity, SD of contrast correlation coefficient |
| | 69 (Echo texture) | Histogram (16)<br>Gray-level co-occurrence matrix, GLCM (22)<br>Gray-level run-length matrix, GLRLM (13) |

| | | |
|---|---|---|
| | | Gray-level size zone matrix, GLSZM (13)<br>Neighborhood gray-tone difference matrix, NGTDM (5) |
| Posterior echo pattern | 11 (Posterior echo distribution) | Relative contrast between posterior region and adjacent tissue (PR vs. AT), Relative brightness contrast between PR vs. AT, Contrast variance between PR vs. AT, Contrast coefficient between PR vs. AT, SD of PR, Mean contrast of PR, contrast SD of PR, Mean covariance of PR, Covariance SD of PR, Mean non-similarity of PR, SD of the non-similarity of PR |
| | 69 (Posterior echo texture) | Same as echo texture |
| Calcification | 11 | Calcification area, Minimum calcification area, Maximum calcification area, Total calcification circumference, SD of roundness, Number of calcification points, SD of calcification area, SD of the circumference of calcification points, Minimum distance between calcification points , Maximum distance between calcification points |
| Wavelet | 412 | Wavelet feature of original image, wavelet feature of margin, boundary, echo pattern, posterior acoustic pattern, calcification |

Three-step feature selection including two-side Wisconsin rank sum test, genetic algorithm and sparse representation was used to select the most relevant features to LNM, and the Support vector machine (SVM) was used to build the LNM diagnosis model.

Supplementary Note 3. Quantitative Evaluation Indexes

Suppose TP, TN, FP, FN represent true positive, true negative, false positive and false negative, then accuracy (ACC), sensitivity (SENS), specificity (SPEC), positive predictive value (PPV), negative predictive value (NPV), and Matthew's correlation coefficient (MCC) can be calculated as:

$$\mathrm{ACC} = \frac{TP+TN}{TP+TN+FP+FN}$$

$$\mathrm{SENS} = \frac{TP}{TP+FN}$$

$$\mathrm{SPEC} = \frac{TN}{TN+FP}$$

$$\mathrm{PPV} = \frac{TP}{TP+FP}$$

$$\mathrm{NPV} = \frac{TN}{TN+FN}$$

$$\mathrm{MCC} = \frac{TP\times TN - FP\times FN}{\sqrt{(TP+FP)(TP+FN)(TN+FP)(TN+FN)}}$$

$$F_1 = 2\cdot\frac{Precision\cdot Recall}{Precision+Recall}$$
