# Supplementary material for: Lymph node metastasis prediction of papillary thyroid carcinoma based on transfer learning radiomics
Source: Nat Commun. 2020 Sep 23;11:4807. doi: 10.1038/s41467-020-18497-3 (PMC7511309; doi:10.1038/s41467-020-18497-3)
Supplement: Supplementary file 2 — Reporting Summary [file 41467_2020_18497_MOESM2_ESM.pdf]

nature research

Corresponding author(s): DBPR NCOMMS-20-10869B

Last updated by author(s): Aug 20, 2020

Reporting Summary

Nature Research wishes to improve the reproducibility of the work that we publish. This form provides structure for consistency and transparency in reporting. For further information on Nature Research policies, see our Editorial Policies and the Editorial Policy Checklist.

Statistics

For all statistical analyses, confirm that the following items are present in the figure legend, table legend, main text, or Methods section.

n/a	Confirmed	
☐	☒	The exact sample size (n) for each experimental group/condition, given as a discrete number and unit of measurement
☐	☒	A statement on whether measurements were taken from distinct samples or whether the same sample was measured repeatedly
☐	☒	The statistical test(s) used AND whether they are one- or two-sided<br>*Only common tests should be described solely by name; describe more complex techniques in the Methods section.*
☒	☐	A description of all covariates tested
☒	☐	A description of any assumptions or corrections, such as tests of normality and adjustment for multiple comparisons
☐	☒	A full description of the statistical parameters including central tendency (e.g. means) or other basic estimates (e.g. regression coefficient) AND variation (e.g. standard deviation) or associated estimates of uncertainty (e.g. confidence intervals)
☐	☒	For null hypothesis testing, the test statistic (e.g. F, t, r) with confidence intervals, effect sizes, degrees of freedom and P value noted<br>*Give P values as exact values whenever suitable.*
☒	☐	For Bayesian analysis, information on the choice of priors and Markov chain Monte Carlo settings
☐	☒	For hierarchical and complex designs, identification of the appropriate level for tests and full reporting of outcomes
☒	☐	Estimates of effect sizes (e.g. Cohen's d, Pearson's r), indicating how they were calculated

Our web collection on statistics for biologists contains articles on many of the points above.

Software and code

Policy information about availability of computer code

Data collection	Three data sets were included in this study. The first two data sets were selected from 3172 patients who underwent thyroid examination from April 2015 to December 2017. Among 3172 patients, 1013 PTC cases with unifocal lesions were used as the main cohort. The second data set contained 368 PTC cases with multifocal lesions, which referred as the independent testing set 1. The third data set was from 1691 patients at two separate hospitals from October 2008 to September 2018. 513 PTC cases of unifocal lesion were selected and referred as the independent testing set 2.
Data analysis	Matlab R2015b; Python 3.5

For manuscripts utilizing custom algorithms or software that are central to the research but not yet described in published literature, software must be made available to editors and reviewers. We strongly encourage code deposition in a community repository (e.g. GitHub). See the Nature Research guidelines for submitting code & software for further information.

Data

Policy information about availability of data

All manuscripts must include a data availability statement. This statement should provide the following information, where applicable:

- Accession codes, unique identifiers, or web links for publicly available datasets
- A list of figures that have associated raw data
- A description of any restrictions on data availability

The data that support the findings of this study are available from the corresponding author upon reasonable request.

Field-specific reporting

Please select the one below that is the best fit for your research. If you are not sure, read the appropriate sections before making your selection.

- [x] Life sciences
- [ ] Behavioural & social sciences
- [ ] Ecological, evolutionary & environmental sciences

For a reference copy of the document with all sections, see nature.com/documents/nr-reporting-summary-flat.pdf

Life sciences study design

All studies must disclose on these points even when the disclosure is negative.

Sample size	4863 patients with ultrasound suspected thyroid lesions were studied in this prospective study from Oct. 2008 to Sep. 2018, and 1894 patients with pathologically confirmed PTC were finally enrolled.
Data exclusions	(1) there were measuring lines on the ultrasound images; (2) The nodules were too large to obtain images covering the complete outline of the nodules even by adjusting the position of the scanning section; (3) patients received preoperative treatment; (4) missing clinical information, such as incomplete ultrasound images for multifocal lesions, incomplete lymph node information.
Replication	The reported results can be reproduced by using our software.
Randomization	The samples in the main cohort were randomly divided into the training cohort and test cohort in cross-valadation.
Blinding	The investigator were blinded to group allocation during data collection and analysis.

Reporting for specific materials, systems and methods

We require information from authors about some types of materials, experimental systems and methods used in many studies. Here, indicate whether each material, system or method listed is relevant to your study. If you are not sure if a list item applies to your research, read the appropriate section before selecting a response.

Materials & experimental systems

n/a	Involved in the study	
x		Antibodies
x		Eukaryotic cell lines
x		Palaeontology and archaeology
x		Animals and other organisms
	x	Human research participants
x		Clinical data
x		Dual use research of concern

Methods

n/a	Involved in the study	
x		ChIP-seq
x		Flow cytometry
x		MRI-based neuroimaging

Human research participants

Policy information about studies involving human research participants

Population characteristics	1894 patients with pathologically confirmed PTC were finally enrolled.
Recruitment	(1) patients underwent thyroid ultrasound diagnosis and had clear B-mode ultrasound images, (2) patients confirmed to be PTC after thyroidectomy. (3) patients received lymph node dissection (LND) and the ground truth of central cervical LNM (sometimes combined with lateral cervical LNM) was according to the pathologic evaluation.
Ethics oversight	This study was approved by the institutional ethics committee of the involved multi-centers.

Note that full information on the approval of the study protocol must also be provided in the manuscript.
